# Coronavirus Disease 2019 (COVID-19) Admission Screening at a Tertiary-Care Center, Iowa 2020

**DOI:** 10.1017/ash.2021.3

**Published:** 2021-07-29

**Authors:** Mohammed Alsuhaibani, Takaaki Kobayashi, Alexandra Trannel, Stephanie Holley, Oluchi Abosi, Kyle Jenn, Holly Meacham, Lorinda Sheeler, William Etienne, Angie Dains, Mary Kukla, Emily Ward, Bradley Ford, Michael Edmond, Melanie Wellington, Daniel Diekema, Jorge Salinas

## Abstract

**Background:** Hospitalized patients may unknowingly carry severe acute respiratory coronavirus virus 2 (SARS-CoV-2), even if they are admitted for other reasons. Because SARS-CoV-2 may remain positive by reverse-transcriptase polymerase chain reaction (RT-PCR) for months after infection, patients with a positive result may not necessarily be infectious. We aimed to determine the frequency of SARS-CoV-2 infections in patients admitted for reasons unrelated to coronavirus disease 2019 (COVID-19). **Methods:** The University of Iowa Hospitals and Clinics is an 811-bed tertiary-care center. We use a nasopharyngeal SARS-CoV-2 RT-PCR to screen admitted patients without signs or symptoms compatible with COVID-19. Patients with positive tests undergo a repeat test to assess cycle threshold (Ct) value kinetics. We reviewed records for patients with positive RT-PCR screening admitted during July–October 2020. We used a combination of history, serologies, and RT-PCR Ct values to assess and qualify likelihood of infectiousness: (1) likely infectious, if Ct values were <29, or (2) likely not infectious, if 1 or both samples had Cts <30 with or without a positive SARS-CoV-2 antinucleocapsid IgG/IgM test or history of a positive result in the past 90 days. Contact tracing was only conducted for patients likely to be infectious. We describe the isolation duration and contact tracing data. **Results:** In total, 6,447 patients were tested on hospital admission for any reason (persons under investigation or admitted for reasons other than COVID-19). Of these, 240 (4%) had positive results, but 65 (27%) of these were admitted for reasons other than COVID-19. In total, 55 patients had Ct values available and were included in this analysis. The median age was 56 years (range, 0–91), 28 (51%) were male, and 12 (5%) were children. The most frequent admission syndromes were neurological (36%), gastrointestinal (16%), and trauma (16%). Our assessment revealed 23 likely infections (42%; 14 definite, 9 possible) and 32 cases likely not infectious (58%). The mean Ct for patients who were likely infectious was 22; it was 34 for patients who were likely not infectious. Mean duration of in-hospital isolation was 6 days for those who were likely infectious and 2 days for those who were likely not infectious. We detected 8 individuals (1 healthcare worker and 7 patients) who were exposed to a likely infectious patient. **Conclusions:** SARS-CoV-2 infection in patients hospitalized for other reasons was infrequent. An assessment of the likelihood of infectiousness including history, RT-PCR Cts, and serology may help prioritize patients in need of isolation and contact investigations.

**Funding:** No

**Disclosures:** None

